# [^68^Ga]Ga-RM2 PET/CT reveals small distant metastases not detected by conventional imaging in primary estrogen receptor-positive breast cancer

**DOI:** 10.1007/s00404-023-06985-y

**Published:** 2023-03-21

**Authors:** Kerstin Michalski, Katharina Müller-Peltzer, Ingolf Juhasz-Böss, Philipp T. Meyer, Juri Ruf, Jasmin Asberger

**Affiliations:** 1 Department of Nuclear Medicine, Medical Center, Faculty of Medicine, University of Freiburg, University of Freiburg, Freiburg, Germany; 2 Department of Nuclear Medicine, University Hospital Würzburg, Würzburg, Germany; 3 Department of Diagnostic and Interventional Radiology, Medical Center, Faculty of Medicine, University of Freiburg, University of Freiburg, Freiburg, Germany; 4 Department of Nuclear Medicine, Klinikum Karlsruhe, Karlsruhe, Germany; 5 Department of Obstetrics and Gynecology, Medical Center, Faculty of Medicine, University of Freiburg, University of Freiburg, Freiburg, Germany

## Presentation

**Fig. 1:**
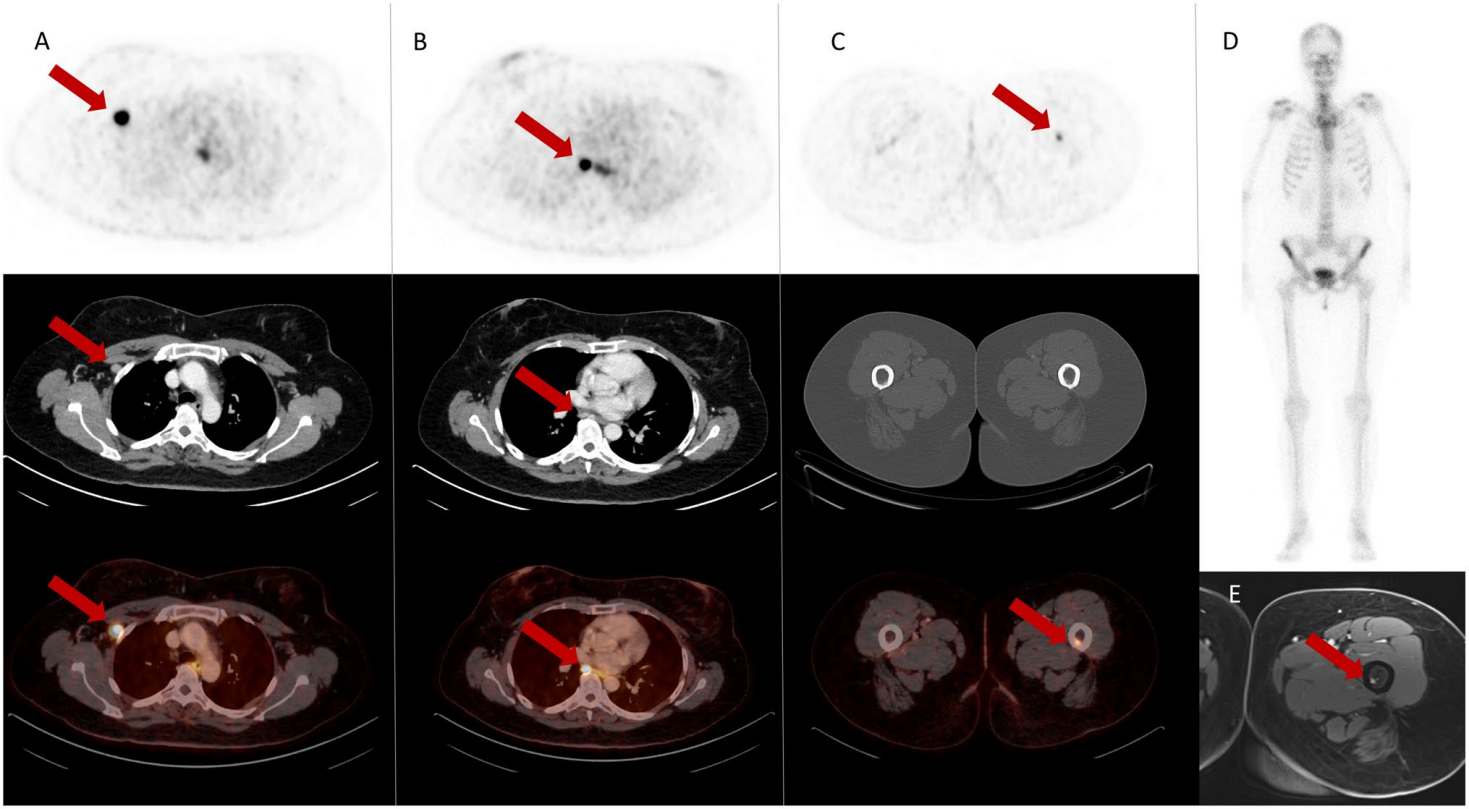
PET/CT imaging with [^68^Ga]Ga-RM2 (A-C; axial slices of PET [top], CT [middle] and fusion images [bottom]). An ipsilateral deep axillary enlarged lymph node with intense tracer uptake was detected, compatible with a local lymph node metastasis **A**. High tracer uptake was observed in a small subcarinal, distant lymph node metastasis **B**, confirmed by biopsy. Furthermore, high focal tracer uptake was found in the left proximal femur **C**. Despite an unsuspicious planar bone scan **D**, magnetic resonance imaging **E** was highly suggestive for a bone metastasis

Here we report the case of a woman with primary estrogen receptor (ER)-positive breast cancer (BC) in which positron emission tomography (PET)/computed tomography (CT) using the gastrin-releasing peptide receptor (GRPR) antagonist [^68^Ga]Ga-RM2 led to an upstaging and change of management by revealing small distant metastases not detected by conventional imaging (Fig. 1)

A 43-year-old patient with newly diagnosed cancer of the right breast (cT2 cN0 Mx; ER > 95%, progesterone receptor > 95%, Her2-neu score: 1, MIB-1 proliferation index 60%) underwent preoperative a [^68^Ga]Ga-RM2 PET/CT imaging on compassionate use basis. The PET scan showed a small subcarinal, distant lymph node metastasis and a highly suspicious lesion in the left proximal femur, both not detected on conventional imaging (CT, MRI and bone scan). Given the upstaging from UICC stage II to stage IV [1], primary breast surgery was postponed. In regards to an oligometastatic concept, the patient underwent neoadjuvant chemotherapy, followed by breast-preserving surgery with sentinel lymph node dissection as well as radiotherapy of the breast, the axilla, the subcarinal lymph node and the femur.

## Discussion

[^68^Ga]Ga-RM2 PET/CT is a promising technique for staging of primary BC with positive ER status [2, 3]. In this case, the PET scan led to an additional metastasis-directed therapy. Thus molecular imaging with [^68^Ga]Ga-RM2 PET/CT might improve risk stratification and, ultimately, prognosis of patients with primary ER-positive BC.

## Data Availability

New imaging techniques can facilitate risk stratification for patients. Molecular imaging with [^68^Ga]-RM2 PET/CT better detected primary metastasis of ER-positive BC in the described case. Therapy was adjusted accordingly. The approriate metastasis-directedtherapy can ultimately be expected to improve the patients prognosis.

## References

[1] Brierley JD GM, Wittekind C, editors. TNM classification of malignant tumours.: John Wiley & Sons; 2017.

[2] Stoykow C, Erbes T, Maecke HR, Bulla S, Bartholoma M, Mayer S (2016). Gastrin-releasing peptide receptor imaging in breast cancer using the receptor antagonist (68)Ga-RM2 And PET. Theranostics.

[3] Zang J, Mao F, Wang H, Zhang J, Liu Q, Peng L (2018). 68Ga-NOTA-RM26 PET/CT in the evaluation of breast cancer: a pilot prospective study. Clin Nucl Med.

